# Rapid learning of object names in dogs

**DOI:** 10.1038/s41598-021-81699-2

**Published:** 2021-01-26

**Authors:** Claudia Fugazza, Attila Andics, Lilla Magyari, Shany Dror, András Zempléni, Ádám Miklósi

**Affiliations:** 1 Department of Ethology, Eötvös Loránd University, Budapest, Hungary; 2 MTA-ELTE ‘Lendület’ Neuroethology of Communication Research Group, Budapest, Hungary; 3 Department of Probability Theory and Statistics, Eötvös Loránd University, Budapest, Hungary; 4 MTA-ELTE Comparative Ethology Research Group, Budapest, Hungary

**Keywords:** Evolution, Psychology

## Abstract

Learning object names after few exposures, is thought to be a typically human capacity. Previous accounts of similar skills in dogs did not include control testing procedures, leaving unanswered the question whether this ability is uniquely human. To investigate the presence of the capacity to rapidly learn words in dogs, we tested object-name learning after four exposures in two dogs with knowledge of multiple toy-names. The dogs were exposed to new object-names either while playing with the objects with the owner who named those in a social context or during an exclusion-based task similar to those used in previous studies. The dogs were then tested on the learning outcome of the new object-names. Both dogs succeeded after exposure in the social context but not after exposure to the exclusion-based task. Their memory of the object-names lasted for at least two minutes and tended to decay after retention intervals of 10 min and 1 h. This reveals that rapid object-name learning is possible for a non-human species (dogs), although memory consolidation may require more exposures. We suggest that rapid learning presupposes learning in a social context. To investigate whether rapid learning of object names in a social context is restricted to dogs that have already shown the ability to learn multiple object-names, we used the same procedure with 20 typical family dogs. These dogs did not demonstrate any evidence of learning the object names. This suggests that only a few subjects show this ability. Future studies should investigate whether this outstanding capacity stems from the exceptional talent of some individuals or whether it emerges from previous experience with object name learning.

## Introduction

Fast mapping, the ability to learn a novel word after a single or very few exposures, as long been discussed as a mechanism that contributes to the rapid lexical acquisition that yields vocabularies of thousands of words in human preschool children^1,2^. Acquisition of words so rapidly in a complex environment is claimed to be a truly human feature of language learning. This view has been challenged by two separate reports of two dogs being able to learn object names after few exposures in an exclusion-based task, in which the dog had to select a novel item among familiar named items, upon hearing a novel name^3,4^. However, these interpretations were not conclusive because those studies did not include the necessary control tests, leaving unanswered the question of whether rapid object name learning is a uniquely human skill. In this report, including two new dogs, we find that rapid object name learning is a robust phenomenon in some individuals under specific social conditions. In line with other reports^5^, we find that it is unlikely that rapid learning occurs during exclusion-based tasks. Evidence of rapid object name learning in dogs provides the basis to develop a new animal model for understanding the evolution of object categorisation based on verbal labels.

In the original studies^3,4^, Rico and Chaser, both border collies, were presented with a novel toy among a set of familiar ones, of which they knew the names, and it was shown that they were able to choose the novel toy upon hearing a novel word via a process of elimination^6^. The observation that Rico and Chaser were able to select this novel toy upon hearing its name for the first time, was taken as evidence of having learned a new object name in few exposures, assuming fast mapping as a mental process. However, the interpretation of this elimination process resulting in fast mapping has been challenged (see e.g.^7–9^). Attending to the name of the novel object and associating it to its physical features may be not needed in order to infer by exclusion that a novel word refers to a novel object among known ones because, in this case, it is enough to attend to the features of the known objects to rule them out. An alternative explanation of this result could be that Rico and Chaser may have chosen based on a process of ‘extended exclusion’^5^. Rico was tested in a retention trial, in which the new object that was rewarded in previous exclusion-based choice trials was placed among familiar objects and new, never rewarded objects. In this case, the dog may have differentiated the last object he was rewarded for, from items of which he already knew the names and also from completely novel items that were never rewarded. This process would not necessarily require learning the name of the object. When the last presented object is tested in the presence of objects of which the dog had already learned the names and in presence of completely novel ones, as it was the case for Rico, after having excluded the objects of which he had previously learned the names, the dog may still have selected the correct object by choosing the only object he had been recently rewarded for, without necessarily having learned its name (see also^10^). This experiment would have been more convincing if two equally newly learned objects had been present, so that the dog would have had to rely on their respective names to make its choice. To exclude the proposed alternative explanation, a subsequent study^5^ applied such more stringent control for testing object name learning in a 12-year-old Yorkshire Terrier named Bailey. Once the dog succeeded in exclusion-based tasks similar to those of the previous studies, two new toys that she had correctly selected during the exclusion-based task were pitted against each other. Bailey was then tested on her choice of one or the other toy upon hearing their names. Bailey failed in this task. This failure may indicate that dogs lack the ability to learn a novel object name after few exposures in general or that this ability does not arise in exclusion-based choice situations. Although it is also not possible to exclude that, while Bailey failed in this task, Rico and Chaser would have succeeded, this control condition was not included in the earlier experiments. Thus, additional studies are needed to test whether an ability to rapidly learn object names is present in dogs and whether it emerges in such condition.

The failure of learning new words during exclusion tasks has been also documented in human children (e.g.^11^). For children too, excluding known items and selecting a novel one upon hearing a novel word does not necessarily imply learning the name of the object^12–14^. For example, when 2- to 4-year-old children were tested on whether they had learned new words during exposure to exclusion-based tasks, less than 50% of them succeeded^11^. Thus, it seems that exposure to novel word-referent in an exclusion task does not necessarily result in learning the name of the presented objects, even in human children. Successful word learning most often occurs through a process by which the child repeatedly attends to, and encodes the features of the novel object (e.g.^13,15^).

It may be argued that exclusion-based tasks do not represent the most common and natural situation for humans (and for dogs) to learn object names. It is known that ostensive naming facilitates word learning in human children by increasing their attention to the target object^16–18^, thus increasing the chances that they would attend to and encode some of its features that can then be associated with its name. Dogs, due to their evolutionary history and development in the human environment, are particularly receptive to human ostensive communicative cues (e.g.^19–22^). Dogs living in a human environment are naturally exposed to daily interactions with their owners that include playing with toys and referring to those using a name and ostensive communication. Therefore, we hypothesised that this more natural social context may allow rapid learning of object names in dogs to emerge, probably by enhancing their attention to the features of the object and to its name. Importantly, the possibility of rapid learning of object names in this context has not been investigated before and, so far, learning object names in dogs emerged only in few individuals and after some form of massive training (e.g.^4,23^). We tested rapid object-name learning in two dogs with previous knowledge of multiple toy-names in two different contexts, a social context and an exclusion-based task similar to those used in the previous studies.

The extant literature about successful object-name learning in dogs only reports about very few subjects and, typically, studies are conducted on only one dog (e.g.^3–5^). Therefore, in an additional test we also aimed at investigating whether most dogs are able learn object names after very few exposures or this skill is only shown by few individuals. For this, we tested with the same procedure rapid object-name learning in 20 typical family dogs. Based on the paucity of subjects reported to have learned object names in previous studies, we hypothesized that this capacity may be present only in some individuals.

To fully consolidate memory of a new object name, a robust memory representation of the name–object association must be formed so that it can eventually be recalled later. For human children, a stable association between the word and its referent typically emerges over a longer period of time (slow-mapping) as the child repeatedly encounters those^13,15^. To test whether memory consolidation of object-names can emerge in dogs after few exposures, we also carried out tests after delays of 10 min and 1 h. We expected that, due to the limited exposure to the objects and their names, the dogs’ memory of those decays rapidly with the longer delays.

## Results and discussion

### Baseline tests and test with the experimenter

The subjects of this study were a 4-year-old female border collie named Whisky and a 9-year-old female Yorkshire terrier named Vicky Nina. The dogs learnt object names during spontaneous unplanned exposure in their daily life while playing with toys with their owners. We first tested their previously acquired vocabulary knowledge of 59 objects for Whisky and 42 objects for Vicky Nina (mostly dog toys) in a *baseline test*, in which the dogs were requested to fetch their familiar toys (choosing between subsets of respectively 16–20 toys for Whisky and 6–10 for Vicky Nina), when their owner spoke the name of the toy: “Bring $\langle$name of the object$\rangle$”. In all tests we controlled for potential cues given by the owner or by the experimenter by keeping the objects out of view from the owner and the experimenter. The dogs were able to select the requested toy (Whisky: 54 out of 59 correct trials, 91.53%; Vicky Nina: 27 out of 42 correct trials, 64.29%; binomial probability p < 0.001 for both dogs), showing that they had learned and consolidated the names of most of the objects during earlier interactions with their owners, in absence of any formal training session. We also tested Whisky with the experimenter, instead of the owner, requesting a subset of her familiar toys (16–20). The dog was able to retrieve the correct toy in 13 trials out of 15 (86.67%)—binomial probability p < 0.001, showing that her acquired knowledge of object names was extended to when the name was spoken with a different voice and with different accent and pronunciation. Previous studies reporting the ability of dogs to retrieve named objects when their names were spoken by a different, unfamiliar person only included one subject^5^. Therefore, our results extend this conclusion to at least another dog.

### Object name learning choice tests

**Figure 1:**
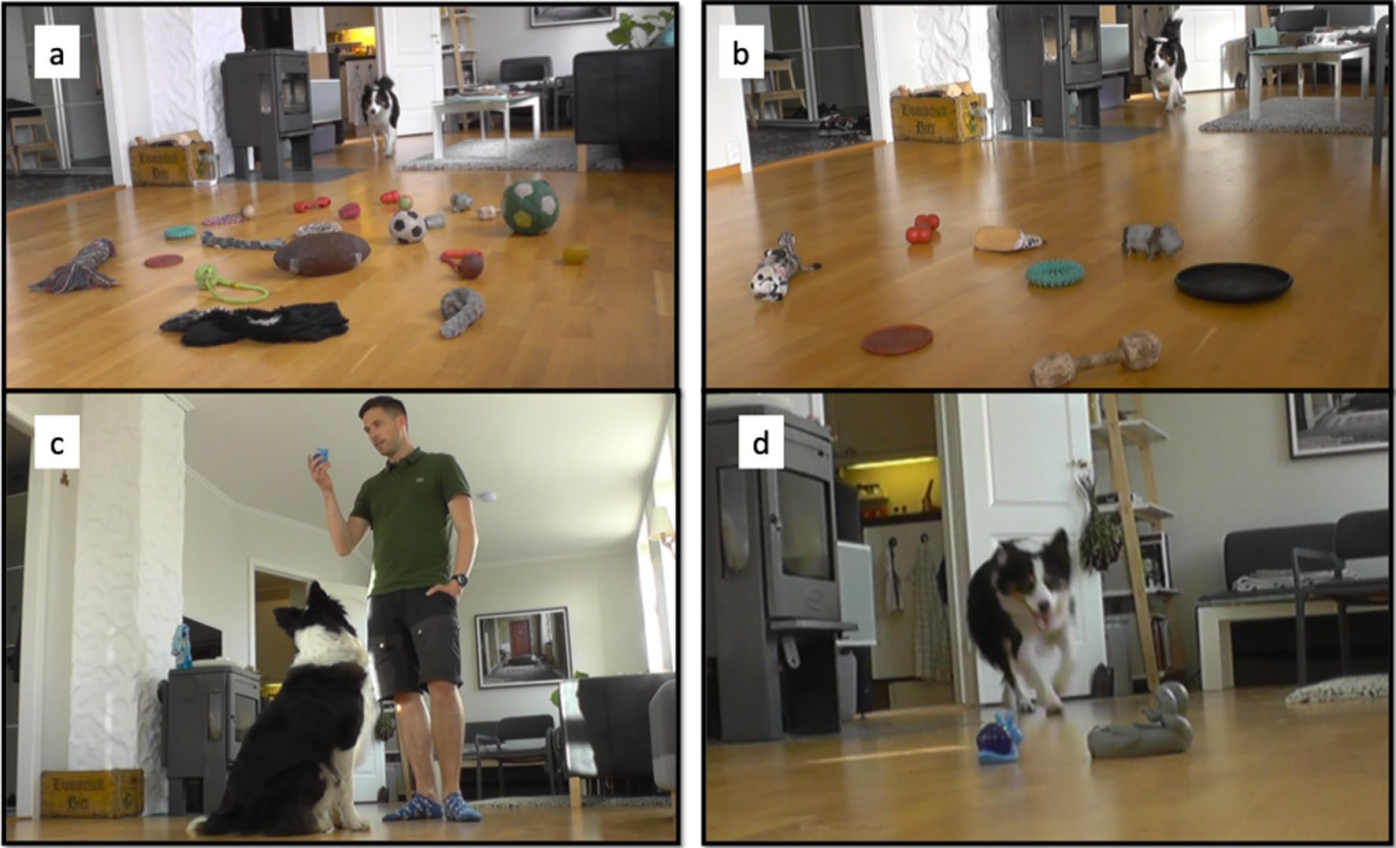
Scenarios of the experiment. (**a**) *Baseline test*: the dog is asked to select a familiar toy among other familiar toys upon hearing its name; (**b**) *Exclusion condition*: the dog is asked to select a new toy among familiar toys upon hearing a new word; (**c**) *Social condition*: the owner shows a new toy to the dog and plays with it while naming it; (**d**) *Choice test*: the dog is asked to select one of two toys to which it was exposed either in the *exclusion condition* or in the *social condition*.

**Figure 2:**
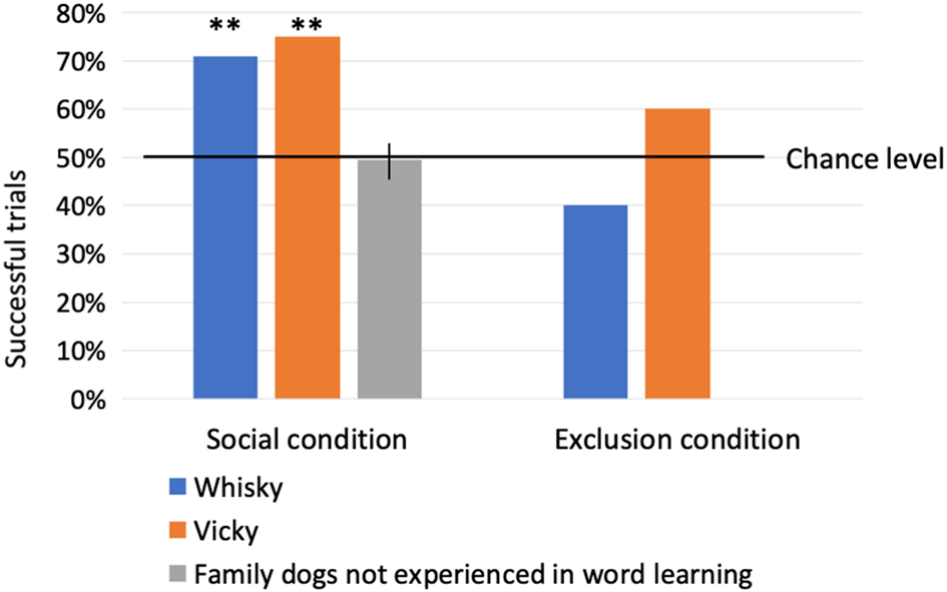
Percentage of correct trials (17 out of 24 for Whisky; 15 out of 20 for Vicky Nina; mean success rate of correct trials for the 20 non-experienced family dogs: 49.8%, 95% bootstrap confidence interval: 46.4; 53.1, based on 480 trials) in the choice test, during which dogs were requested to choose one of two objects they had been exposed to in a social context (social condition) and during an exclusion-based task (exclusion condition). **Indicate performance significantly above chance level (p < 0.05).

We then exposed the dogs to the pairing of novel names to novel objects in two different conditions: (1) in an ostensive-social context, where the owner showed the two novel objects and pronounced the two novel names (one at a time), while playing with the toy with the dog—*social condition*—and 2) in an exclusion-based task similar to those used in previous studies on fast mapping^3,4^, where the two novel objects were among seven familiar ones (one at a time) and the owner uttered a novel name to request the novel object—*exclusion condition*. As rapid learning, by definition, should occur after very few exposures, in both conditions the dogs were exposed to only four repetitions of the novel object name, before being tested on their learning outcome of those novel object names. Two minutes after the exposure to the pairing of a novel name with a novel object for two objects in one condition, the dogs underwent an *object name learning choice test*, in which only those two toys were present—always placed out of view from the owner—and the dogs had to select between those upon hearing their respective names (Fig. 1). This method allowed us to test for the true learning of the name of the objects because the dogs could not rely on exclusion of already known or never-seen toys^9^, as only the two newly presented toys were present. Thus, to select the named toy, the dogs had to recall the encoded features of the two objects, together with their names—or, at least, of one of them and then choose the other based on exclusion. Whisky and Vicky Nina retrieved the correct toy significantly above chance in the choice test after exposure to the social condition (Whisky: 17 correct trials out of 24, 70.84%; Binomial probability p = 0.032; Vicky Nina: 15 correct trials out of 20, 75%; Binomial probability p = 0.02; Fig. 2), but not in the choice test after exposure in the exclusion condition (Whisky: 8 correct trials out of 20, 40%; Binomial probability p = 0.868; Vicky Nina: 12 correct trials out of 20, 60%; Binomial probability p = 0.252; Fig. 2), although Whisky invariably succeeded in selecting the novel toys upon hearing a novel name during the exclusion-based task (40 trials out of 40, 100% correct trials) and Vicky Nina did so above chance (21 out of 40 correct trials, 52.5%; Binomial probability p < 0.001). This observation supported earlier similar findings^3,4^ but importantly, those studies did not include any control for extended exclusion-based choice to test whether the subjects had really learned the object names (see above and^5^). Whisky and Vicky Nina chose the appropriate object in exclusion-based trials, but their success was not based on learning that the word was a name for a specific object. Similar negative results were also shown in chimpanzees^24^ rhesus monkeys^25^ and sea lions^10^. In contrast, ostensive exposure to the pairing between a novel object and its name in the social condition allowed rapidly learning object names in these two dogs.

These results provides evidence of rapid learning of new object names after very few exposures in a non-human species, thereby challenging the view that this is a uniquely human feature of language learning. We suggest that, during the brief social interaction with their owners, when the owner spoke the name of the object while showing it and playing with it, the dogs focused their attention on both the name and the features of the object and thus they were able to encode and then recall those in the subsequent choice test. This ostensive social context facilitated object name acquisition to a greater extent than being exposed to the pairing of the novel name with the novel object in the exclusion-based task. During the exposure in the social condition, the joint attention and the play with the object may have contributed to learning about the features of the objects to be associated with their names. Therefore, as expected, dogs’ receptivity to human ostensive communicative cues (e.g.^19–22^) facilitated learning in this condition. In contrast, it is likely that in exclusion-based tasks, similarly to human infants (e.g.^11^), dogs may have merely attended to the known objects, which helped in ruling them out and selecting the remaining novel one upon hearing a novel name. In this condition, the presence of the familiar toys may also have distracted the attention of the dogs. This suggests that, as opposed to ostensive naming^16^, during exclusion-based choice, attention is deployed in a non-optimal manner for learning to occur, as it is mainly targeted to the familiar objects, to rule them out. Indeed, it seems that learning by exclusion is not likely to be the preferential mechanism through which rapid word learning occurs in children either^26^. Teaching words in a socially rich context—although after longer training—has also been shown to boost learning in phylogenetically distant species, i.e. parrots (^27,28^ for review), chimpanzees and bonobos^29^.

The social condition of this study was devised to resemble as much as possible to natural spontaneous interactions occurring typically between dogs and owners, when the owners play with their dogs with toys, naming the toys and using ostensive communication. Therefore, the situation presented in the social condition was more familiar, had a shorter duration and was probably less cognitively demanding than the exclusion condition and these factors may also have facilitated learning in these dogs. Moreover, in the social condition the dog was exposed to the object and to its name four times while in the exclusion condition the dogs had to select the novel item and, if it erred, it received potentially fewer correct exposures than in the social situation, hence less opportunity for learning. However, learning did not occur for Whisky either, even though she never erred in the exclusion-based task. Since the two conditions differ in several respects, we do not aim at directly comparing them. We rather aimed at testing object name learning with methods similar to those used in previous studies (i.e., exclusion-based tasks) and in conditions that are arguably more similar to the social contexts in which human infants learn words (social context). We do not aim at drawing a parallel between learning in humans and dogs, nor we claim a direct parallel between the context in which human infant learn words and our social condition. Nevertheless, our results do show that rapid learning of object names in a social context is not restricted to humans.

We also note that the strict criterion for testing object name learning adopted in this study forced us to test learning of two object names at once, which may be intrinsically more demanding—and hence, lead to lower performance in the tests—compared to learning one object name at a time.

### Object name learning choice tests with typical dogs

To investigate whether the ability of learning object names in very few exposures in a social, ostensive context is restricted to dogs that have already shown the ability to learn object names, we tested 20 typical family dogs in six object name learning choice tests of four trials each—identical to those administered to Whisky and Vicky Nina—after exposure to novel object names in the social condition. We selected dogs whose owners reported that they were motivated to fetch toys. As these dogs did not already know object names, they could not be tested in the exclusion condition. These dogs were recruited among owners and dog trainers who volunteered for the study and were representative of the population of typical family dogs. We included only dogs whose owners reported that the dog was motivated to play with toys. Most of them had received some basic or advanced training, but they had no reported previous knowledge of object-names.

Typical dogs' performance was used as a baseline to judge the performance of Whisky and Vicky Nina. As typical dogs' performance was poor and had low variability (10–13 of the 24 trials for every dog, with an average success rate of 49.8%), the usual assumption of the binomial distribution was not applicable. Also, there was a high proportion of dogs that chose the same toy in all trials (resulting in 2 of 4 successful trials in every *object name learning choice test*). We estimated the p-value of Whisky’s and Vicky Nina’s performance (respectively 17 of 24 and 15 of 20 correct trials) by a bootstrap simulation approach based on the pool of typical dogs, using a complex model and found that their result differed significantly from that of typical dogs (p = 0.004, see “Data analysis” for details on the complex model and the bootstrap simulation approach used).

Different factors may have contributed to the typical dogs’ failure to learn object names in few exposures, including insufficient attentional focus to the physical features of objects and to the names, eventually resulting from limited experience about the relevance of those stimuli. The results showing that the typical dogs most often chose the same object are also in accordance with dogs’ tendency to develop a preference, whenever they make frequent errors^30,31^. The current data does not allow to disentangle the role of previous experience with object-name learning and individual differences in the capacity to learn that objects can have names. If the poor performance of typical dogs is confirmed by future research exposing dogs to more extensive repetitions of the object names, then it would indicate that only a smaller population of dogs possess the skill to represent object names in a way comparable to humans, which would also explain the very limited number of dogs reported in the literature to know object names. These exceptional capacities may be the result of genetic and/or developmental variation in complex behavior potentially due to non-heritable “developmental noise” in brain wiring^32^. This may provide an interesting scenario for investigating the cognitive mechanisms of object name learning in a non-human species. Alternatively, and/or additionally, these results may also suggest that previous extensive experience of learning object names may be necessary for rapid learning abilities to emerge in dogs^33^.

### Memory decay choice tests

Vicky Nina and Whisky had learned and could remember the newly learned object names for at least two minutes after the exposure to the new object names in the social context, when the *object learning name choice test* was administered. To test memory consolidation, whenever they succeeded in at least three trials out of four in a given *object learning name choice test* of the social condition, we tested the dogs after longer delays: another choice test was administered after 10 min from the previous one (*Memory decay choice test*). In case of successful performance in at least three trials out of four in this 10-min-delay test, another test was administered after 1 h. In the *object learning name choice tests*, Whisky had succeeded in selecting the correct toy in at least three trials out of four in five choice tests out of six and Vicky Nina in four choice tests out of five. Thus, Whisky was tested in five *memory decay choice tests* after 10-min-delay and Vicky Nina in four. In these *memory decay choice tests* after 10 min-delay, Whisky succeeded to select the correct toy in at least three trials out of four in two tests and Vicky Nina in one. In the *memory decay choice tests* after 10 min-delay, Whisky succeeded in total 11 trials out of 20 (55% trials; Binomial probability p = 0.252); Vicky Nina succeeded in total 9 trials out of 16 (56.25% trials; Binomial probability p = 0.402). Since Whisky succeeded in at least three trials out of four in two *memory decay choice tests* after 10 min-delay and Vicky in one, they were tested respectively in two and one *memory decay choice tests* after 1 h-delay. After the 1 h-delay, both dogs succeeded in 50% of the trials (Whisky: four trials out of 8; Vicky Nina: two trials out of four; Binomial probability, respectively, p = 0.637; p = 0.687). The smaller amount of data collected after the longest delays may have limited the sensitivity of the test. However, the dogs’ poor performance in tests after these longer delays suggests that, while in the present case the dogs could retain in their memory the new object names for at least two minutes, consolidation of new object names is rather a gradual process, probably involving experiencing the statistical regularity of the co-occurrence of a name and its referent across multiple encounters in time^14,15,34^. Slow consolidation has been reported in human children as well (e.g.^26,35^).

This study provides evidence of a non-human mammalian species learning object names after very few (four) exposures and developing a short-term memory that lasts for at least a few (at least two) minutes. Our results also suggest that this rapid learning occurs in a social context. Although we cannot exclude that Rico and Chaser had learned the names of the objects during the exclusion-based task, our rigorous method suggests that rapid object name learning may not typically occur in exclusion-based contexts.

Dogs, because of their natural development in the human environment, which includes extensive exposure to words and objects, may constitute an ideal model species, more suitable than captive animals raised in artificial laboratory conditions, to study the evolution of the mechanisms underlying language learning. These results constitute a preliminary step into establishing such a non-human model.

The fact that Whisky and Vicky Nina differed significantly from the pool with their higher success rate also raises the question of whether most dogs, if provided with extensive experience of object name learning during early puppyhood, would develop skills similar to those displayed by Whisky and Vicky Nina or whether these two dogs, and other sporadic individuals reported in the literature (e.g.^3–5,36^), have an atypical ability to learn object names and can learn the concept that objects have names. The very limited number of dogs that are documented to be able to learn multiple object names makes the findings of the studies on those few individuals extremely valuable as they can highlight commonalities and differences with humans and consequently offer a viable way to investigate the evolution of language, concerning receptive communication.

## Methods

### Subjects

The subjects of this study were two family dogs: Whisky, a 4-year-old female border collie living with her owners in Norway and Vicky Nina, a 9-year-old Yorkshire terrier living with her owners in Brazil. The owners of both dogs reported to us via personal communication that the dogs were motivated to play with toys since puppyhood, thus they provided them with toys for play and named them. The owners reported also that, to attribute names to the toys, they would pronounce each name several times in a playful context, allowing the dogs to take the toy in their mouth and asking the dogs to fetch it, then playing again with it, while naming it again. This way, Whisky and Vicky Nina reportedly learned the name of over 59 (Whisky) and 42 (Vicky Nina) different objects, including plush toys, rubber and plastic toys of various shapes, balls, frisbees, knotted ropes and animals of various materials. Each of these toys had been given a proper name (e.g. ‘rat’, ‘fox’ etc.) in Norwegian by Whisky’s owners and in Portuguese by Vicky Nina’s owner. Before the tests, the owners provided a list of all of the named toys. This allowed us to choose novel toys for the object name learning tests. Additionally, we tested (only in the *object name learning choice test* of the social condition) N = 20 typical family dogs that did not have such knowledge of multiple object names (Age: 4.45 ± 1.9 years; n = 12 female and n = 8 male; Breeds: 3 Golden retrievers, 3 Australian Kelpies, 2 Border Collies, 2 Australian shepherds, 1 miniature Schnauzer, 1 Groenendal, 1 Belgian Malinois, 1 Tervueren, 1 Boxer, 1 Labrador retriever, 1 Dutch Shepherd, 1 Vizsla, 1 German shepherd, 1 mongrel).

### Testing procedure

The tests were carried out in a place that was familiar to each dog (their house or training club). For Whisky, the tests were carried out at her house in Bergen (Norway). For Vicky Nina the tests were carried out in a hotel room in Saõ Paulo (Brazil). Vicky Nina spent a whole day and night in this room with her owner before the tests started, to ensure that she had the opportunity to become familiar with the place. The typical dogs were tested in areas familiar to them: either their house or their training centre.

For all the subjects, before the tests started, the toys were laid on the floor in an area not visible to the owner (an adjacent room for Whisky, behind the wall of a corridor, out of the owner’s view for Vicky Nina and behind the back of the owners for the other dogs tested). This way the owners, not knowing where the objects were, could not inadvertently guide the dogs’ choices with other bodily cues when pronouncing the name of the toys. Both the experimenter and the owner remained out of view of the toys during the test trials. The owners asked the dogs to retrieve a named toy from that position and waited there for the dog to appear with a toy in its mouth. The dog was free to move in the area but was not allowed to approach the area where the toys were, before a request to bring a named toy was made (owners could encourage the dogs to stay there by using food rewards). When a request was uttered, the dogs went to the toys, selected one and brought it back to the owner, leaving it at his/her feet or nearby (see Video 1). We considered a choice to have been made when the dog appeared with a toy in its mouth.

### Baseline: retrieving known toys based on their verbal names (‘names’)

We first tested Whisky’s and Vicky Nina’s knowledge of the proper name of all the toys available, to which their owners had given a name (59 toys for Whisky and 42 toys for Vicky Nina) by asking them to fetch all of them, one by one, upon hearing their names. During the test, the owner requested the toys from the out-of-view area, while 16–20 (Whisky) and 6–10 (Vicky Nina) randomly chosen toys were laid on the floor of the adjacent area—thus out of view from the owner—by the experimenter. After every 5 trials the experimenter took to the area another set of 5 randomly chosen toys, so that the number of toys the dogs could choose from always varied from 16 to 20 (Whisky) or from 6 to 10 (Vicky Nina). The number of toys laid on the floor differed for the two dogs due to more limited availability of space for the toys in the case of Vicky Nina. (For the statistical analysis the strictest chance level was used, as if there were always 16 and 6 objects present to choose from, respectively for Whisky and Vicky Nina).

### Test protocol

- The randomly chosen toys were laid by the experimenter on the floor in the out-of-view area at approximately 20 cm from one another.
- The owner and the dog stayed out of view of the toys.
- After positioning the toys, the experimenter reached the owner in the out of view area.
- The owner asked the dog to bring one of the toys (predetermined on a random basis) by saying “bring $\langle$name of the object$\rangle$”.
- If the dog brought the correct toy, it was verbally praised and received a treat.
- If the dog brought the wrong toy, the choice was scored as incorrect and the dog was not praised.
- All the toys were requested once (59 trials for Whisky; 42 trials for Vicky Nina).

### Test with the experimenter

For Whisky, the procedure described above was repeated with a subset of 15 randomly chosen toys, but this time the experimenter, instead of the owner, asked for the toys and interacted with the dog when she brought the toys. Not only was the experimenter a different person, speaking with a different voice, but also her accent and pronunciation of the words may have been quite different because she is Italian while Whisky’s owners are Norwegian and have always spoken to the dog in Norwegian. This test consisted of 15 trials with 15 randomly chosen objects from those used in the baseline test.

Due to the limited time of availability of Vicky Nina’s owner, this test was not carried out with Vicky Nina.

### Object name learning choice tests

Whisky and Vicky Nina were tested on their ability to learn a new name referring to a toy in two different conditions: (1) social context (*social condition*) and (2) process of exclusion (*exclusion condition*). The test to assess whether they had learned the new names, an *object name learning choice test*—see below—was carried out after the exposure to two new toys in each condition (Table 1). This allowed us to pit two toys, to which the dogs had been just exposed, against each other, therefore excluding that the dogs could still choose based on exclusion of familiar toys. The *object name learning choice test* was identical for the two conditions. The tests of the two conditions were carried out in a randomized order.

**Table 1:** Testing procedure.

Social condition and *object name learning choice test*	Exclusion condition and *object name learning choice test*
(1) Playful exposure to toy A	(1) Exclusion-based task for toy C
(2) Playful exposure to toy B	(2) Exclusion-based task for toy D
(3) 2 min pause	(3) 2 min pause
(4) Choice test: between A and B	(4) Choice test: between C and D

The table illustrates the testing procedure used for testing Whisky and Vicky Nina.

The 20 dogs that did not have extensive knowledge of object names were tested in the social condition with the same procedure used for Whisky and Vicky Nina.

### Social condition

In the *social condition*, the dogs were exposed to the pairing of the novel name with the novel toy in a social context with their owner. The owner was instructed to show the toy to the dog and play with it with the dog while saying the name of the toy four times during this brief playful interaction. After this was done consecutively for two toys with two different names, the dogs were exposed to a *object name learning choice test*, where we tested their ability to select the correct toy between the two newly introduced ones, upon hearing its name (see below). Since rapid learning, by definition, should occur after very few exposures, we kept the number of trials for every novel toy pair to only four trials (two per novel object, in a randomized order), thereby minimizing the possibility that the dogs would learn the object names during the test.

Overall, we ran 6 *object name learning choice test* with two different novel toys in each test in the social condition for Whisky, 5 *object name learning choice test* for Vicky Nina (due to limited availability of the owner) and 6 *object name learning choice test* for the other 20 typical family dogs.

### Social condition—exposure to the names of the toys

- The experimenter gave a novel toy to the owner and s/he decided how to name it. S/he was asked to choose a name that did not resemble any already known name of other toys.
- The owner took the toy in his/her hand, called the dog’s attention by calling its name and showed the toy to the dog by saying a sentence meaning “look, this is $\langle$name of the object A$\rangle$” in a happy and playful tone of voice.
- Then the owner played with this toy with the dog, allowing the dog to mouth it and tossing it on the floor, so that the dog could take it and play with it.
- During this free interaction, the owner pronounced the name of this toy another 3 times (e.g., while tossing it on the floor, the owner said “get $\langle$name of the object A$\rangle$” and when the dog took it “this is $\langle$name of the object A$\rangle$”).
- This interaction was free in the sense that the owner could play how s/he wanted, so that this resembled as much as possible a natural interaction of the kind that occurred usually between the owner and the dog when they played with toys.
- The number of times the name of the toy was spoken was restricted to four in total.
- This procedure was carried out for two novel toys (e.g. A and B) consecutively, then the dog received an *object name learning choice test* on these two newly introduced toys.

### Object name learning choice test

The choice test was carried out ~ 2 min after the dog was exposed to two new toys in the social or in the exclusion condition (see below). The protocol was identical, irrespective of the condition in which the dog was exposed to the pairing of the names to the toys.

The experimenter laid the two newly introduced toys on the floor of the adjacent area at approximately 20 cm from one another then returned to where the owner and the dog were (i.e., out of view from the toys).

The owner and the dog stayed out of view of the toys during the choice test trials.

When the experimenter walked back to the owner, the owner asked the dog to bring one of the toys (predetermined by a semi randomized schedule) by saying “bring $\langle$name of the object$\rangle$”.

- If the dog brought the correct toy, it was verbally praised and received a treat.
- If the dog brought the wrong toy, the choice was scored as incorrect and the dog was not praised.
- The toy that the dog retrieved was immediately taken back to the living room by the experimenter at every trial.
- Every *object name learning choice test* consisted of four trials.
- The order of the requests of the two novel toys was semi-randomized so that each of them was requested two times in a testing session.
- The position of the toys in every test was determined on a random basis.
- Sample photos of the objects and their names are provided in Fig. S1.

### Exclusion condition

In the *exclusion condition*, Whisky and Vicky Nina were exposed to an exclusion-based task session with a new toy requested four times followed by another similar exclusion-based task session with another new toy, also requested four times. These two new toys were then pitted against each other in the subsequent *object name learning choice test*.

This way, every pair of exclusion-based task session was followed by a choice test with the two newly introduced toys.

The mean duration of the exclusion-based session was 3.15 ± 1.34 min.

Overall, in the Exclusion condition we exposed Whisky and Vicky Nina to 10 exclusion-based task tests with 2 novel toys and 7 familiar toys each, resulting in 5 pairs of new toys to be used in the subsequent 5 *object name learning choice tests*.

### Exclusion condition—exposure to the names of the toys

The experimenter gave a novel toy to the owner and the owner decided how to name it. S/he was asked to choose a name that did not resemble any already known name of other toys.

The experimenter laid 7 familiar (already known) toys randomly selected from those successfully retrieved in the Baseline and the novel one on the floor of the adjacent area, at approximately 20 cm from one another.

Then the experimenter went back to the owner and the owner asked the dog to bring one of the familiar named toys by saying “bring $\langle$name of the object$\rangle$”.

The owner and the dog stayed out of view of the toys.

If the dog brought the correct toy, it was praised verbally and received a treat.

If the dog brought the wrong toy, its choice was scored as incorrect and it was not praised.

The owner asked for the different (old and novel) toys based on a predetermined randomized schedule.

To control for novelty preference, we always asked for one, two or three—randomly determined—familiar toys before asking for the novel one.

When asking for the novel toy, the owner repeated the same procedure as above but said the novel name (e.g. “Bring $\langle$name of the object C$\rangle$”).

After every three trials, the experimenter placed back the toys that the dog had brought.

In every session the novel toy was requested four times, among other trials in which familiar toys were requested.

Once an exclusion-based session was carried out for two novel toys consecutively (e.g. C and D), the dog received an *object name learning choice test* on these two newly introduced toys.

Overall 10 exclusion-based sessions were carried out and, consequently, 5 *object name learning choice test* after exposure to exclusion-based tasks were carried out.

### Memory decay choice tests

Whenever Whisky and Vicky Nina were successful in at least 3 trials out of 4 in a given choice test, they received a similar test after a 10 min retention interval. This test was identical to the preceding one, except that the semi-randomized order of the requested toys differed. If the dog was successful in at least 3 trials out of 4 in this 10-min-retention choice test, it was tested after 1 h. During the retention interval, the dog was let free in the area in the absence of the toys. Given the poor performance of the typical family dogs without object name knowledge, the tests with delays longer than two minutes were not administered to them.

### Data analysis

**Figure 3:**
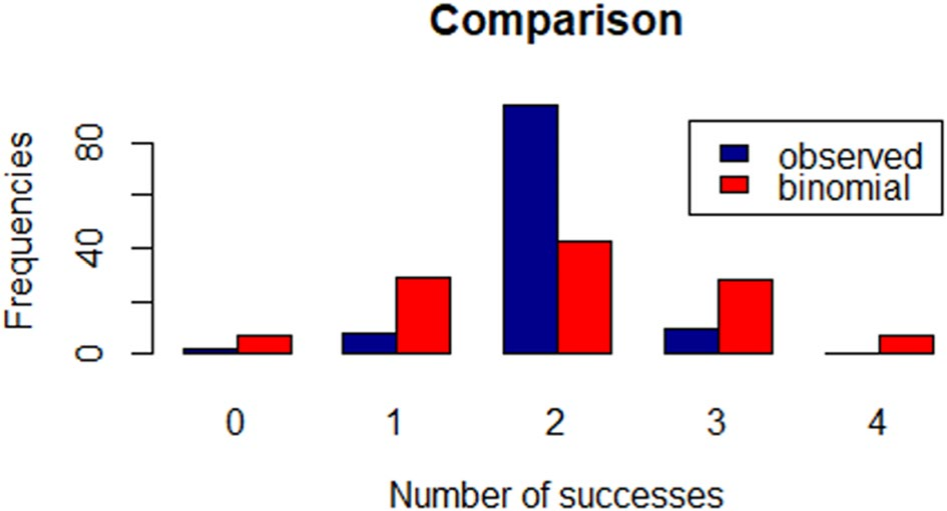
Comparison of the observed distribution of successes (out of 4 trials) of the N = 20 typical dogs in the choice tests with the fitted binomial distribution.

- For the binomial probability calculations of correct choices in every test, we set chance level based on how many objects were laid on the floor, from which the dog could choose.
- For Whisky, in the baseline and in the test with the experimenter, we calculated chance level conservatively at 0.06, as if there were always 16 toys to choose from (while they ranged between 16 and 20). For Vicky Nina we calculated chance level conservatively at 0.17, as if there were always 6 toys to choose from (while they ranged between 6 and 10).
- We also calculated the performance of the dogs when retrieving the new toys during the exclusion-based task. Here, there were from 8 to 5 toys from which the dogs could choose. Thus, we set chance level conservatively at 0.2, as if there were always only 5 toys to choose from.
- In the choice test, there were always two objects that the dogs could select, thus, chance level was set at 0.5.
- To account not only for correct choices but also for the behaviour of “choosing always the same toy”, frequently observed in the typical dogs, we applied a special model (see Fig. 3 for the histogram of the successes, together with the fitted binomial distribution as a proof of this phenomenon: we have many more “2 out of 4” cases than expected). In this model we assumed two disjoint possibilities:

  - in the given test the dog brings back always the same toy, resulting in exactly two successes out of four, let us assume that this happens with the probability q;
  - (b) with probability 1 − q, the dog will bring random toys, resulting in a binomial distribution for the successes. The observed frequencies (blue columns of Fig. 3) allowed us to estimate a probability q for case a) and the success probability p of the binomial model, as follows. The expected total number of correct choices for a dog, performing k tests under this model is
    $$ T = q \cdot 2 \cdot k + (1 - q) \cdot 4p \cdot k $$
    as it is always 2 when it chooses the same toy and 4*p* for the random case (*k* was chosen as 6 in the experiment), while the expected proportion of having exactly 2 successes out of four trials in a test is
    $$ P_{2} = q + (1 - q) \cdot \left( {\begin{array}{*{20}c} 4 \\ 2 \\ \end{array} } \right)p^{2} (1 - p)^{2} . $$

We have equated these theoretical values with their empirical counterparts and solved the equations for *q* (the chance for choosing always the same toy) and *p* (the success probability in the binomial model). The estimated values were q = 0.57 and p = 0.495—the latter one is in accordance with our hypothesis of p = 0.5.

As there is dependence across the trial (implied by the fact that the same dogs performed the experiment 6 times) and there is no closed form for this type of distributions, we used bootstrap simulations from the original pool of nontrained dogs, to get repetitions for the estimators. We estimated the p and q values for N = 500 bootstrap samples and simulated 100 repetitions of “virtual trials” based on these estimators. The resulting 50,000 samples allowed for a reliable estimation of the p-value for Whisky’s and Vicky Nina’s performance, which turned out to be less than 0.01. The ideas behind these considerations can be found in^37^.

### Informed consent

Informed consent was obtained from the owners of the dogs to publish identifying information and images in an online open-access publication.

Informed consent was obtained from the owners of the animals involved in the study, for their participation.

### Ethical statement

All experiments were performed in accordance with relevant guidelines and regulations for the care and use of animals during research. All methods were carried out in accordance with relevant guidelines and regulations for human participants. The Institutional Committee of Eötvös Loránd University has approved the experiments of this study (N. PE/EA/691-5/2019).

## Supplementary Information

Supplementary Video 1.

Supplementary Information.

## References

[1] Carey S, Wanner E, Gleitman L (1982). Semantic development: The state of the art. Language Acquisition: The State of the Art.

[2] Carey S (2010). Beyond fast mapping. Lang. Learn. Dev..

[3] Kaminski J, Call J, Fischer J (2004). Word learning in a domestic dog: Evidence for “fast mapping”. Science.

[4] Pilley JW, Reid AK (2011). Border collie comprehends object names as verbal referents. Behav. Process..

[5] Griebel U, Oller DK (2012). Vocabulary LEARNING in a Yorkshire Terrier: Slow mapping of spoken words. PLoS ONE.

[6] Halberda J (2003). The development of a word-learning strategy. Cognition.

[7] Bloom P (2004). Can a dog learn a word?. Science.

[8] Markman EM, Abelev M (2004). Word learning in dog?. Trends Cogn. Sci..

[9] Kaulfuss P, Mills DS (2008). Neophilia in domestic dogs (*Canis familiaris*) and its implication for studies of dog cognition. Anim. Cogn..

[10] Kastak CR, Schusterman J (2002). Sea lion and equivalence expanding classes by exclusion. J. Exp. Anal. Behav..

[11] Wilkinson KM, Ross E, Diamond A (2003). Fast mapping of multiple words: Insights into when “the information provided” does and does not equal “the information perceived”. Appl. Dev. Psychol..

[12] Riches NG, Tomasello M, Conti-Ramsden G (2005). Verb learning in children with SLI: Frequency and spacing effects. J. Speech Lang. Hear. Res..

[13] Horst JS, Samuelson LK (2008). Fast mapping but poor retention by 24-month-old infants. Infancy.

[14] Mather E, Plunkett K (2009). Learning words over time: The role of stimulus repetition in mutual exclusivity. Infancy.

[15] Smith LB, Yu C (2008). Infants rapidly learn word-referent mappings via cross-situational statistics. Cognition.

[16] Axelsson EL, Churchley K, Horst JS (2012). The right thing at the right time: Why ostensive naming facilitates word learning. Front. Psychol..

[17] Baldwin DA, Markman EM, Bill B, Desjardins RN, Irwin JM (1996). Infants’ reliance on a social criterion for establishing word–object relations. Child Dev..

[18] Dunham PJ, Dunham F, Curwin A (1993). Joint attentional states and lexical acquisition at 18 months. Dev. Psychol..

[19] Bensky MK, Gosling SD, Sinn DL (2013). The world from a dog’s point of view: A review and synthesis of dog cognition research. Adv. Stud. Behav..

[20] Topál J, Gergely G, Erdohegyi A, Csibra G, Miklósi A (2009). Differential sensitivity to human communication in dogs, wolves, and human infants. Science.

[21] Tauzin T, Csik A, Kis A, Kovacs K, Topál J (2015). The order of ostensive and referential signals affects dogs' responsiveness when interacting with a human. Anim. Cogn..

[22] Kupán K, Miklósi Á, Gergely G, Topál J (2010). Why do dogs (*Canis familiaris*) select the empty container in an observational learning task?. Anim. Cogn..

[23] Fukuzawa M, Mills DS, Cooper JJ (2005). More than just a word: Non-semantic command variables affect obedience in the domestic dog (*Canis familiaris*). Appl. Anim. Behav. Sci..

[24] Beran MJ (2010). Use of exclusion by a chimpanzee (Pan troglodytes) during speech perception and auditory—Visual matching-to-sample. Behav. Process..

[25] Paxton Gazes R, Chee NW, Hampton RR (2018). Monkeys learn but do not choose through exclusion. Anim. Behav. Cogn..

[26] Bion RAH, Borovsky A, Fernald A (2013). Fast mapping, slow learning: Disambiguation of novel word-object mappings in relation to vocabulary learning at 18, 24, and 30 months. Cognition.

[27] Pepperberg IM (1994). Vocal learning in African Grey parrots: Effects of social interaction. Auk.

[28] Pepperberg IM (2010). Vocal learning in Grey parrots: A brief review of perception, production, and cross-species comparisons. Brain Lang..

[29] Brakke KE, Savage-Rumbaugh ES (1995). The development of language skills in bonobo and chimpanzee-I. Comprehension. Lang. Commun..

[30] Nitzschner M, Kaminski J, Melis A, Tomasello M (2014). Side matters: Potential mechanisms underlying dogs’ performance in a social eavesdropping paradigm. Anim. Behav..

[31] Gácsi M, Kara E, Belényi B, Topál J, Miklósi Á (2009). The effect of development and individual differences in pointing comprehension of dogs. Anim. Cogn..

[32] Linneweber GA, Andriatsilavo M, Dutta SB, Bengochea M, Hellbruegge L, Liu G, Ejsmont RK, Straw AD, Wernet M, Hiesinger PR, Hassan BA (2020). A neurodevelopmental origin of behavioral individuality in the Drosophila visual system. Science.

[33] Ramos D, Mills DS (2019). Limitations in the learning of verbal content by dogs during the training of object and action commands. J. Vet. Behav..

[34] Axelsson EL, Horst JS (2014). Contextual repetition facilitates word learning via fast mapping. Acta Psychol..

[35] McMurray B, Horst JS, Samuelson LK (2012). Word learning as the interaction of online referent selection and slow associative learning. Psychol. Rev..

[36] Kaminski J, Tempelmann S, Call J, Tomasello M (2009). Domestic dogs comprehend human communication with iconic signs. Dev. Sci..

[37] Rice JA (2007). Mathematical Statistics and Data Analysis.

