# Supplementary material for: Rapid learning of object names in dogs
Source: Sci Rep. 2021 Jan 26;11:2222. doi: 10.1038/s41598-021-81699-2 (PMC7838202; doi:10.1038/s41598-021-81699-2)
Supplement: Supplementary file 2 — Supplementary Information. [file 41598_2021_81699_MOESM2_ESM.docx]

**Supplementary** **information for**

**Rapid learning of object names in dogs**

Claudia Fugazza^1*^, Attila Andics^1,2*^, Lilla Magyari^1,2^, Shany Dror^1^, András Zempléni^3^

and Ádám Miklósi^1,4^

^1^*Department of Ethology, Eötvös Loránd University, Budapest*

*^2^ MTA-ELTE ‘Lendület’ Neuroethology of Communication Research Group*

^3^*Department of Probability Theory and Statistics, Eötvös Loránd University, Budapest*

*^4^ MTA-ELTE Comparative Ethology Research Group*

*** Corresponding author:** Claudia Fugazza and Attila Andics

**Email:** [claudia.happydog@gmail.com](mailto:claudia.happydog@gmail.com), attila.andics@gmail.com

**Supplementary material and methods**

Whisky was tested in 3 different testing occasions, one in April 2018, one in August 2018 and one in October 2018. Due to the limited availability of the owner and the long trip involved, Vicky Nina was tested in 5 days in November 2018. Therefore, for Vicky Nina, intervals ranging between 30 min and 24 hours elapsed between tests; for Whisky the intervals could range between 30 min and several months.

The tests with the dogs that did not have extensive vocabulary knowledge were carried out in Hungary and in Israel between November 2018 and March 2020.

The (Norwegian) names of the pairs of new toys used for Whisky in the exclusion condition were the following:

1. Dumbo – Snegeln
2. Bubu – Kikinen
3. Snor – Ledning
4. Skolen – Tellerken
5. Trakt – Aba
6. Putin - Kadaffi

The (Portuguese) names of the pairs of new toys used for Vicky Nina in the exclusion condition were the following:

1. Onza – Morango
2. Corazao – Shit
3. Orsu – Coroja
4. Kiki – Ben
5. Melmen - Bulldog

The (Norwegian) names of the pairs of new toys used for Whisky in the social condition were the following:

1. Delfin - Lion
2. Purse – Baby duck
3. Koppen – Gaffel
4. Torsken - Lady bug
5. Tomateske - Avocadobox

The (Portuguese) names of the pairs of new toys used for Vicky Nina in the social condition were the following:

1. Ippopotamo – Bear
2. Star – Flamingo
3. Pao – Pinky
4. Rena – Poney
5. Noel - Cane

**Supplementary description of the results**

Novelty preference in the exclusion-based task:

Whisky never retrieved the novel toy in the first trials of the exclusion-based task, when the owner asked for familiar toys.

Vicky Nina retrieved the new toy instead of the requested familiar one in the first trials of 4 testing sessions out of 10.

Supplementary Figure S1.

**Figure S1.**

Photos of samples of the new objects used in the object label learning tests and their names.
